# Hormone replacement therapy and false positive recall in the Million Women Study: patterns of use, hormonal constituents and consistency of effect

**DOI:** 10.1186/bcr1364

**Published:** 2005-12-23

**Authors:** Emily Banks, Gillian Reeves, Valerie Beral, Diana Bull, Barbara Crossley, Moya Simmonds, Elizabeth Hilton, Stephen Bailey, Nigel Barrett, Peter Briers, Ruth English, Alan Jackson, Elizabeth Kutt, Janet Lavelle, Linda Rockall, Matthew G Wallis, Mary Wilson, Julietta Patnick

**Affiliations:** 1 National Centre for Epidemiology and Population Health, Australian National University, ACT 0200, Australia; 2 Cancer Research UK Epidemiology Unit, University of Oxford, Richard Doll Building, Roosevelt Drive, Oxford OX3 7LF, UK; 3 Breast Screening Service, Princess of Wales Community Hospital, Stourbridge Road, Bromsgrove B61 0BB, UK; 4 West of London Breast Screening Service, Charing Cross Hospital, Fulham Palace Road, London W6 8RF, UK; 5 Gloucestershire Breast Screening Service, Linton House, Thirlestaine Road, Cheltenham, Glos GL53 7AS, UK; 6 The Breast Care Unit, Oxford Radcliffe Hospital NHS Trust, The Churchill Hospital, Old Road, Headington, Oxford OX3 7JH, UK; 7 Patricia Massey Breast Screening Unit, Queen Alexandra Hospital, Cosham, Portsmouth, Hants PO6 3LY, UK; 8 Avon Breast Screening, Central Health Clinic, Tower Hill, Bristol BS2 0JD, UK; 9 North Lancashire Breast Screening Service, Royal Lancaster Infirmary, Ashton Court, Lancaster LA1 4GG, UK; 10 The West Sussex Breast Screening Service, Worthing Hospital, Park Avenue, Worthing, West Sussex BN11 2DH, UK; 11 Breast Screening Unit, Coventry and Warwick Hospital, Stoney Stanton Road, Coventry CV1 4FH, UK; 12 Greater Manchester Breast Screening Service, The Nightingale Centre, Withington Hospital, Nell Lane, Manchester M20 0PT, UK; 13 National Health Service Cancer Screening Programmes, Fulwood House, Old Fulwood Road, Sheffield S10 3TH, UK

## Abstract

**Introduction:**

Current and recent users of hormone replacement therapy (HRT) have an increased risk of being recalled to assessment at mammography without breast cancer being diagnosed ('false positive recall'), but there is limited information on the effects of different patterns of HRT use on this. The aim of this study is to investigate in detail the relationship between patterns of use of HRT and false positive recall.

**Methods:**

A total of 87,967 postmenopausal women aged 50 to 64 years attending routine breast cancer screening at 10 UK National Health Service Breast Screening Units from 1996 to 1998 joined the Million Women Study by completing a questionnaire before screening and were followed for their screening outcome.

**Results:**

Overall, 399 (0.5%) participants were diagnosed with breast cancer and 2,629 (3.0%) had false positive recall. Compared to never users of HRT, the adjusted relative risk (95% CI) of false positive recall was: 1.62 (1.43–1.83), 1.80 (1.62–2.01) and 0.76 (0.52–1.10) in current users of oestrogen-only HRT, oestrogen-progestagen HRT and tibolone, respectively (*p* (heterogeneity) < 0.0001); 1.65 (1.43–1.91), 1.49 (1.22–1.81) and 2.11 (1.45–3.07) for current HRT used orally, transdermally or via an implant, respectively (*p* (heterogeneity) = 0.2); and 1.84 (1.67–2.04) and 1.75 (1.49–2.06) for sequential and continuous oestrogen-progestagen HRT, respectively (*p* (heterogeneity) = 0.6). The relative risk of false positive recall among current users appeared to increase with increasing time since menopause, but did not vary significantly according to any other factors examined, including duration of use, hormonal constituents, dose, whether single- or two-view screening was used, or the woman's personal characteristics.

**Conclusion:**

Current use of oestrogen-only and oestrogen-progestagen HRT, but not tibolone, increases the risk of false positive recall at screening.

## Introduction

Around one-third of women attending the UK National Health Service Breast Screening Programme (NHSBSP) from 1996 to 2000 were using hormone replacement therapy (HRT) at the time and about 16% were former users [1]. Women undergoing mammography are at a higher risk of being recalled to assessment with no subsequent diagnosis of breast cancer ('false positive recall') if they are current users of HRT, compared to women who are not currently using HRT [2,3]. Among past users of HRT, the elevated risk of false positive recall diminishes significantly with increasing time since last use of HRT but still appears to be elevated among women ceasing use within the last five years [3]. Details regarding the effect of different patterns of use of HRT on the risk of false positive recall, including the effect of duration of use of HRT and use of different HRT preparations, are lacking, and it is not clear whether the effects of current and past use of HRT vary between women with different characteristics and between studies.

This study investigates in detail the effects of different patterns of use of HRT on the risk of false positive recall, as well as the consistency of HRT effects. It also presents a meta-analysis of results from studies examining the effect of current and past use of HRT on false positive recall.

## Methods

At the time of the study, all 50 to 64 year old women in the UK who were registered with a general practitioner were invited to attend the NHSBSP approximately once every three years for routine mammographic screening. The NHSBSP is aimed at screening asymptomatic women, whereas women with symptoms are encouraged to consult their general practitioner.

Women recruited into the Million Women Study (described in detail elsewhere [4]) who attended screening at ten selected breast screening units (Avon, Gloucestershire, Hereford and Worcester, Manchester, North Lancashire, Oxfordshire, Portsmouth, Warwickshire Solihull and Coventry, West of London and West Sussex) from June 1996 to the end of March 1998, were followed in detail for their outcome at screening. The women received a study questionnaire accompanying their invitation and were asked to return the completed form when they attended for screening. The questionnaire requested information regarding lifestyle and socio-demographic factors, reproductive factors, past health and use of HRT, and asked for signed consent for follow-up. The recruitment questionnaire can be viewed at the study website [5]. At the time of recruitment, nine of the ten screening centres used two-view mammography at the first screening round and single-view mammography at subsequent rounds; one centre used two-view mammography for all screens.

### Definitions

The variables for these analyses, including use of HRT, were defined according to what was reported on the recruitment questionnaire, that is, at baseline. Women were asked which specific proprietary preparation of HRT they had used most recently, and for these analyses the preparations are grouped according to the hormonal constituents of each preparation listed in the British National Formulary [6]. Women whose periods had ceased either naturally or as the result of a bilateral oophorectomy are defined as postmenopausal. Women aged 55 and over who had had a hysterectomy without oophorectomy were also defined as postmenopausal, as were women aged 55 and over who began use of HRT before their natural menopause [7].

An outcome of screen-detected breast cancer was defined as histologically confirmed screen-detected breast cancer (invasive cancer or carcinoma *in situ*), according to the pathology information recorded by the screening centre. 'False positive recall' was defined, according to screening centre records, as being recalled to assessment following initial screening mammography, without a subsequent diagnosis of screen-detected breast cancer during that screening episode. Women with technically inadequate films that needed to be repeated were classified according to the results of their repeat mammogram.

### Analysis

These analyses include 87,967 postmenopausal women aged 50 to 64 years who did not report a past history of cancer (except non-melanoma skin cancer) at recruitment. (This includes a small number (n = 2,527, 2.9%) who were aged 49 or 65 years when screened, but were close to their 50th or 65th birthday, since women are invited to screening according to their year of birth, rather than their exact age.)

Relative risks (RR) are estimated as odds ratios and 95% confidence intervals (CI) calculated by conditional logistic regression, stratifying by screening centre, age at screening (50 to 52, 53 to 55, 56 to 58, 59 to 61, 62 to 64), time since menopause (<5 years, 5 to 9 years, ≥10 years), reported previous breast screening (no, yes), body mass index (<25 kg/m^2^, ≥25 kg/m^2^) and history of previous breast surgery for a condition other than breast cancer (no, yes). All adjustment factors include an 'unknown' category. Adjustment for other potential confounding factors, such as deprivation index, educational attainment, family history of breast cancer, age at first birth and parity, had no material effect on the findings and are, therefore, not included in the analyses. All tests for trend by duration and time since last use of HRT were calculated using the appropriate median duration or time since last use in each category. Where results are presented graphically, relative risks are shown as black squares with areas inversely proportional to the variance of the log relative risk, indicating the amount of statistical information available for that particular estimate. The corresponding confidence intervals are shown as horizontal lines.

### Meta-analysis

Articles presenting original epidemiological data on the effect of current and past use of HRT on the risk of false positive recall were located through searches of PubMed, the Science Citation Index and through searching reference lists of located references. Information on the relative risk of false positive recall in current and past users of HRT compared to never users was extracted and presented graphically. The summary relative risk was calculated as the exponent of the average of the log relative risks for the relevant studies, weighted according to the inverse of the variance of the individual log relative risks.

## Results

Among 87,967 participating postmenopausal women, 3,043 (3.5%) were recalled to assessment and 3,028 attended this assessment; 15 women (0.02%) were lost to follow-up due to having defaulted attendance at assessment and/or having chosen to be investigated outside the National Health Service. Overall, 399 (0.5%) women were diagnosed with screen-detected breast cancer, leaving 2,629 (3.0%) women with false positive recall.

### Use of HRT and screening outcome

Table 1 shows the proportion of women experiencing false positive recall according to use of HRT, along with the associated relative risk estimates and their confidence intervals. Stratifying by screening centre, age, time since menopause, previous breast screening, body mass index and previous breast surgery, women who had ever used HRT had a significantly higher risk of false positive recall, compared to women who had never used HRT (RR 1.47, 95% CI 1.36–1.60), and this relative risk was particularly elevated among current users (RR 1.64, 95% CI 1.50–1.80) (Table 1). Women who had used HRT in the past were also at a significantly increased risk of false positive recall compared to never users (RR 1.21, 95% CI 1.06–1.38) and this relative risk differed significantly from that in current versus never users of HRT (χ^2^_1_(heterogeneity) = 13.7, *p* < 0.001). There was a significant trend in recall according to time since stopping use of HRT (χ^2^_1_(trend) = 14.0, *p* < 0.001), suggesting the wearing off of the effect of HRT over time in past users [3].

Among current users of HRT, the relative risk was significantly elevated in all duration groups, including those women who had been using HRT for less than one year, and there was no significant trend in the relative risk according to duration of use (Table 1). In contrast, there was a significant trend of increasing risk of false positive recall with increasing duration of use in past users (Table 1). Recent users of HRT are more likely to have used it for long durations, and there were insufficient data to separate out reliably the effects of duration of use and time since last use among past users of HRT.

**Figure 1:**
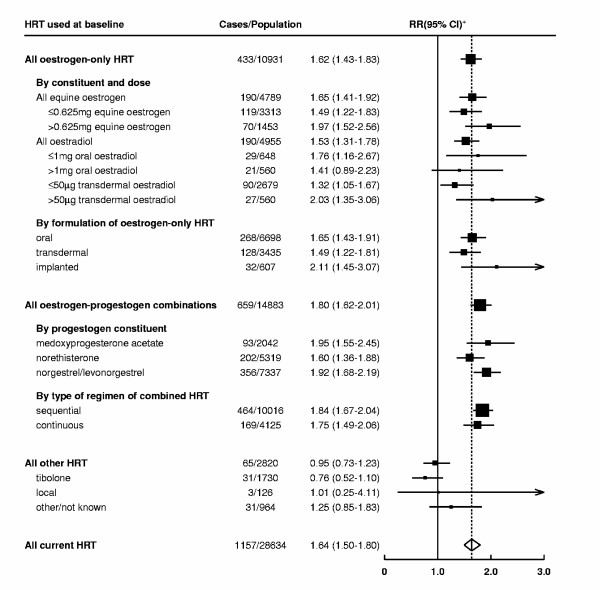
Relative risk (RR) of false positive recall in current versus never-users of hormone replacement therapy (HRT), by preparation currently used. *Stratified by screening centre, age, previous screening, body mass index and previous breast operation. Relative risks are shown as black squares with areas inversely proportional to the variance of the log relative risk, indicating the amount of statistical information available for that particular estimate. The corresponding confidence intervals (CI) are shown as horizontal lines. An arrow is used where the upper confidence limit exceeds the scale of the figure. The diamond represents the overall relative risk and 95% CI of false positive recall in current versus never-users of HRT.

The relative risk of false positive recall differed significantly according to whether a woman was currently using oestrogen-only, oestrogen-progestagen or other types of HRT: RR (95% CI) 1.62 (1.43–1.83), 1.80 (1.62–2.01) and 0.95 (0.73–1.23), respectively (χ^2^_2_(heterogeneity) = 22.2, *p* < 0.0001) (Fig. 1). This was mainly because the relative risk (95% CI) of false positive recall among current users of tibolone was 0.76 (0.52–1.10) compared to women who had never used HRT, and the difference in the relative risk of false positive recall between women currently using oestrogen-only, oestrogen-progestagen and tibolone was highly significant (χ^2^_2_(heterogeneity) = 21.0, *p* < 0.0001). There was no significant difference in the relative risk of false positive recall between women using oestrogen-only and oestrogen-progestagen HRT (χ^2^_1_(heterogeneity) = 2.3, *p* = 0.1) [3].

Among women currently using oestrogen-only or oestrogen-progestagen HRT, there was no significant difference observed according to the type of oestrogen (for conjugated equine oestrogen versus oestradiol, χ^2^_1_(heterogeneity) = 0.5, p = 0.5) or type of progestogen (for medroxyprogesterone acetate versus norethisterone versus norgesterel/levonorgesterel, χ^2^_2_(heterogeneity) = 4.3, *p* = 0.1) in use, according to the dose of oestrogens currently being used (for conjugated equine oestrogen ≤0.625 mg versus >0.625 mg, χ^2^_1_(heterogeneity) = 3.0, *p* = 0.08; for oestradiol ≤1 mg versus >1 mg, χ^2^_1_(heterogeneity) = 0.5, *p* = 0.5; for oestradiol ≤50 μg versus >50 μg, χ^2^_1_(heterogeneity) = 3.4, *p* = 0.07) or according to whether the oestrogen was taken orally, via a patch or an implant (χ^2^_2_(heterogeneity) = 2.8, *p* = 0.2) (Fig. 1). There was no significant difference in the relative risk of false positive recall between those using sequential and continuous oestrogen-progestagen HRT (χ^2^_1_(heterogeneity) = 0.3, *p* = 0.6).

### The consistency of the effect of HRT

**Figure 2:**
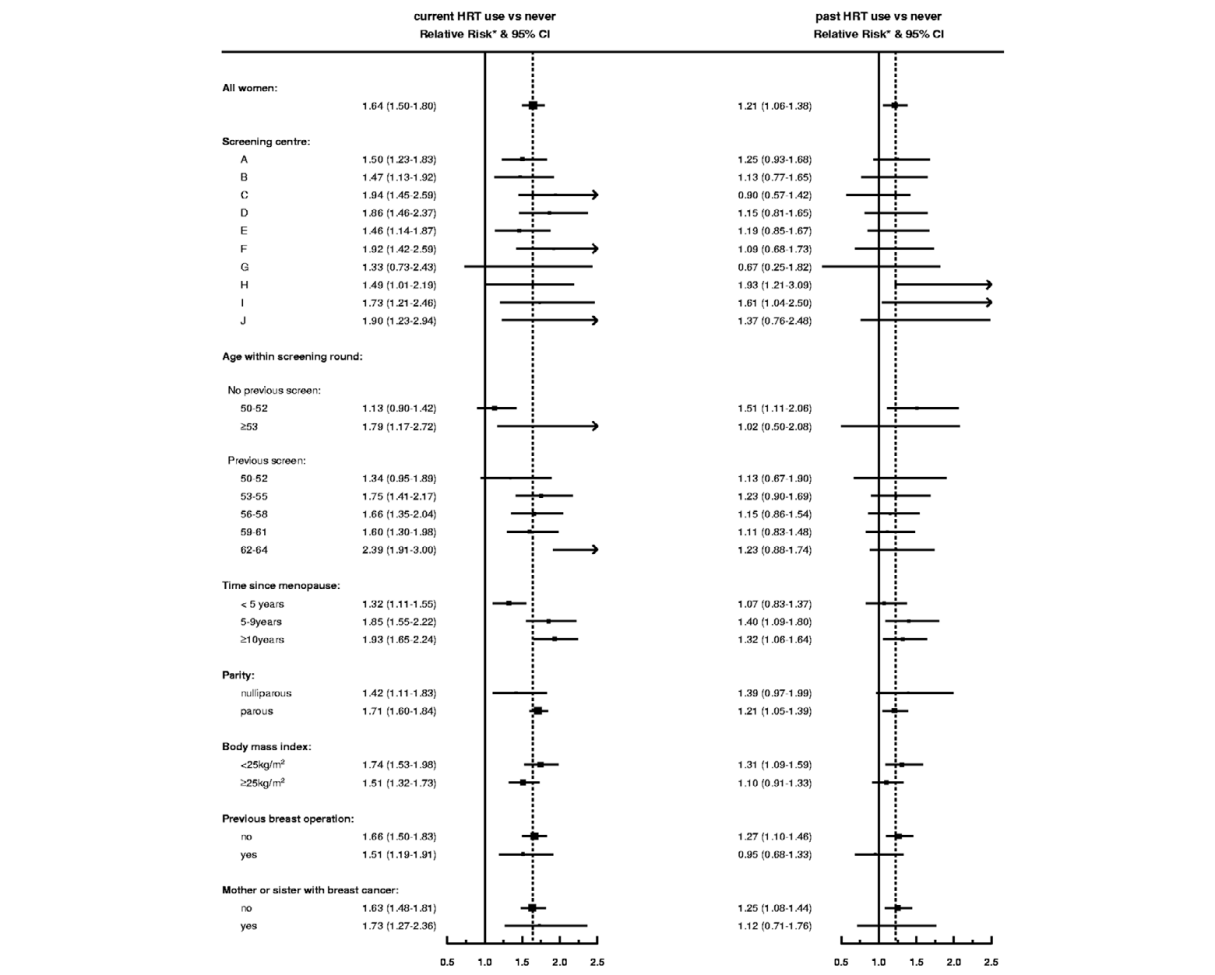
Relative risk (RR) of false positive recall in relation to hormone replacement therapy (HRT) use, in various subgroups of women. *Relative to never-users, stratified by screening centre, age at screening, previous screening, body mass index, previous breast operation and time since menopause (where appropriate). Relative risks are shown as black squares with areas inversely proportional to the variance of the log relative risk, indicating the amount of statistical information available for that particular estimate. The corresponding confidence intervals (CI) are shown as horizontal lines. An arrow is used where the upper confidence limit exceeds the scale of the figure.

To examine how consistent the effects of current and past use of HRT are across other possibly relevant factors, the adjusted relative risks of false positive recall and 95% confidence intervals in current and past users of HRT compared to never users are presented (Fig. 2). Significant heterogeneity was observed in the effect of current use of HRT (global χ^2^_20_(heterogeneity) = 38.2, *p* = 0.01), which was chiefly the result of variation according to the closely related factors of age within screening round and time since menopause. Although this heterogeneity appeared to be greater for time since menopause than for age, there were insufficient data to investigate their dual effects. There was no significant heterogeneity in the effect of HRT according to whether or not women had had a previous screen, after accounting for age. No significant heterogeneity was present for current use of HRT for any other factors examined, nor was significant heterogeneity found for the effect of past use of HRT (global χ^2^_20_(heterogeneity for past use) = 18.1, *p* = 0.6). These included comparisons between the different screening centres (Fig. 2), in that there was no significant difference between the nine centres using single-view mammography among previously screened women (centres A to E and G to J) and the centre using two-view mammography among previously screened women (centre F) (χ^2^_1_(heterogeneity for current use) = 1.1, *p* = 0.3; χ^2^_1_(heterogeneity for past use) = 0.2, *p* = 0.7).

**Figure 3:**
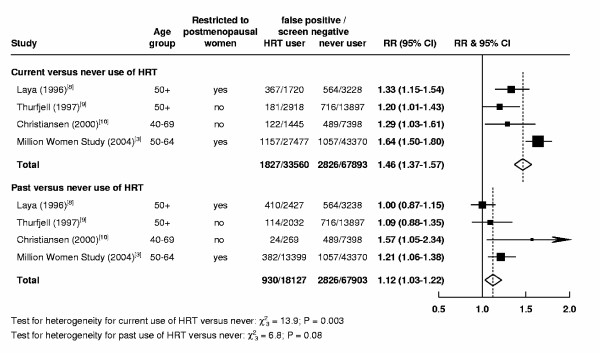
Published results on the relative risk (RR) of false positive recall in relation to use of hormone replacement therapy (HRT). Test for heterogeneity for current use of HRT versus never, χ^2^_3 _= 13.9, *p* = 0.003. Test for heterogeneity for past use of HRT versus never χ^2^_3 _= 6.8, *p* = 0.08. Relative risks are shown as black squares with areas inversely proportional to the variance of the log relative risk, indicating the amount of statistical information available for that particular estimate. The corresponding confidence intervals (CI) are shown as horizontal lines. An arrow is used where the upper confidence limit exceeds the scale of the figure. The diamonds represent the summary relative risks and 95% CIs of false positive recall in current and past versus never-uses of HRT.

Figure 3 summarises the results of this study, along with previous studies that have compared the risk of false positive recall among current and past users of HRT separately with that for never users [8-10]. The overall results show a significantly elevated risk of false positive recall among current users of HRT compared to never users, and a lesser, but still significant, increase in risk among former users. There is significant heterogeneity in the results for current use of HRT but not for past use.

## Discussion

Women who have ever used HRT are at an increased risk of false positive recall compared to never users. The relative risk is greatest in current users and, among past users, declines significantly with increasing time since ceasing use [3]. The relative risk of false positive recall was elevated to a similar extent in users of oestrogen-only and oestrogen-progestagen HRT. The risk of false positive recall in women using tibolone did not differ significantly from that in never users.

The study described here is large and population-based, meaning that major effects of HRT on false positive recall can be quantified and generalised to the screened population in the UK. Detailed prospective data were gathered on the use of HRT immediately prior to screening, including information on current and past use, along with the type (that is, the hormonal constituents) and dose of the preparation currently being used. These data have shown excellent agreement with general practice prescription records [11]. It should be noted, however, that the study had limited power to test for differences in the effect of HRT according to the number of mammographic views in use by different screening centres. Previous results from the Million Women Study have shown that false positive recall is increased in women who are premenopausal, those without a previous NHSBSP mammogram, those reporting previous breast surgery, those who are nulliparous and those with a lower body mass index [7]. The availability of prospectively gathered information on a range of demographic, reproductive and lifestyle factors, including carefully ascertained and classified data on menopause, allowed consideration of these and other important potential confounding factors [1,7].

Current use of HRT was associated with increased false positive recall soon after use commenced. This is consistent with three previous comparable publications, which all show significant increases in the risk of false positive recall with current use of HRT (Fig. 3). There is, however, significant heterogeneity between studies in the magnitude of this effect between studies. The two studies with the lowest relative risks included premenopausal and perimenopausal women [9,10] and not being able to account for this confounding factor [7] may have diluted the findings. Differences in time since menopause among study participants may also have differed between studies. Furthermore, there are large variations in recall rates in different screening programmes, reflecting differences in diagnostic thresholds and other factors, which may also impact on the size of the effect of HRT. The Women's Health Initiative randomised controlled trial found significantly elevated risks of false positive recall among women randomised to oestrogen-progestagen HRT compared to placebo from the first year following randomisation onwards, further supporting the findings of increased false positive recall soon after commencing use of HRT [12].

The effect of current use of HRT was not significantly related to duration of use and, among women using oestrogen alone or combined oestrogen/progestogen, did not differ significantly according to the type of oestrogen and/or progestogen in current use, according to the dose of oestrogen currently in use or according to the mode of delivery of the oestrogen. Two previous smaller studies with data on false positive recall and use of oestrogen-only and oestrogen-progestagen HRT also report no substantive differences in the risk between users of these types of HRT [8,9]; one of these studies suggested possible differences in false positive recall between other HRT regimens, based on small numbers of events, and called for more extensive research in this area [9]. Findings regarding false positive recall in users of tibolone have not, to our knowledge, been reported before.

Overall, former users of HRT had a significantly lower risk of false positive recall than current users but a significantly higher risk than never-users. Although results from previous studies were apparently inconsistent, the totality of the evidence to date shows an increased risk of false positive recall in past users of HRT without significant variation between studies (Fig. 3). The relative risk of false positive recall diminished following cessation of use, but was still significantly raised in the first few years after stopping use [3]. That the risk of false positive recall increases with increasing duration of use in past but not current users of HRT is difficult to interpret. It is not possible to disentangle the effects of duration of use and time since last use in the data and larger studies are required to investigate whether the effect of long duration use of HRT on false positive recall may take longer to wear off than the effect of short duration use.

There is evidence to suggest that women who are currently using oestrogen-only and oestrogen-progestogen HRT have a higher proportion of their mammograms occupied by relatively radiodense tissue, compared to women who have never used HRT [13-15] and this remains a plausible reason for the increase in false positive recall in users. The effects of the different types of HRT on false positive recall seen here, however, are not consistently explained by their observed effects on mammographic density. Most studies find increases in mammographic density in users of combined oestrogen-progestagen and lesser increases in users of oestrogen-only HRT [12,16-22] (although the largest randomised trial investigating the latter, the oestrogen-only arm of the Women's Health Initiative [23], is yet to report on mammographic density), but no significant differences are observed in this study between the effects of oestrogen-progestagen and oestrogen-only HRT on false positive recall [3]. Tibolone appears to have little, if any, effect on mammographic density [24-28], consistent with the lack of a significant effect on false positive recall seen here.

Being recalled to assessment is a stressful experience for most women [29,30], and investigation of women who are ultimately found to have a false positive screen represents a considerable use of resources for screening programmes [31]. Minimisation of recall rates is, therefore, desirable. At the same time, recall rates must be high enough to ensure adequate cancer detection and increased recall may be justified if it results in increased detection of breast cancer. For women who use HRT, who are at an elevated risk of breast cancer compared to non-users [12,32], this issue is of particular importance. Women who use HRT are at an increased risk of having a cancer diagnosed in the interval following a negative screen (an interval cancer) [2,33,34], suggesting that the increase in recall in users is not accompanied by improved detection. The lack of any significant difference in the effect of HRT between screening centres using single-view and two-view mammography suggests that the more widespread adoption of two-view mammography [35] may not affect the elevated risk of false positive recall in women using HRT.

## Conclusion

Current use of oestrogen-only and oestrogen-progestagen HRT, but not tibolone, increases the risk of false positive recall at screening. The elevated risk of false positive recall in users of oestrogen-only and oestrogen-progestagen HRT was observed consistently among the different HRT preparations in use.

## Abbreviations

CI = confidence interval; HRT = hormone replacement therapy; NHSBSP = National Health Service Breast Screening Programme; RR = relative risk.

## Competing interests

The authors declare that they have no competing interests.

## Authors' contributions

Emily Banks, Gillian Reeves, Valerie Beral and Julietta Patnick had the original idea for the study, with important input on practical aspects of study design from Diana Bull, Barbara Crossley, Elizabeth Hilton and Moya Simmonds. Emily Banks and Diana Bull analysed the data and Emily Banks, Gillian Reeves, Valerie Beral and Julietta Patnick interpreted the data. Stephen Bailey, Nigel Barrett, Peter Briers, Ruth English, Alan Jackson, Elizabeth Kutt, Janet Lavelle, Linda Rockall, Matthew Wallis and Mary Wilson contributed to local study design and conduct. All the authors participated in drafting the paper and gave final approval of the version to be published. Emily Banks, Gillian Reeves, Valerie Beral and Diana Bull are guarantors for the study.

## Figures and Tables

**Table 1:** Breast cancer screening outcome in postmenopausal women for users of HRT compared to never users

	Total women screened (n = 87967^a^)	Women recalled to assessment				Adjusted RR (95% CI) of false positive recall^b^	Heterogeneity/trend, *p*-value
		No breast cancer (false positive recall)		Screen-detected breast cancer			

		%	n	%	n		

Total	87,967	3.0	2,629	0.5	399		
							
Never used HRT^c^	44,620	2.4	1,057	0.4	193	1.00	χ^2^_1 _(heterogeneity) = 90.1,
Ever used HRT	43,332	3.6	1,572	0.5	206	1.47 (1.36–1.60)	P < 0.001
							
Past user of HRT	13,829	2.8	382	0.3	48	1.21 (1.06–1.38)	
							
Time since last use (median)							χ^2^_1 _(trend) = 14.0, *p* < 0.001
Current user (0 years)	28,788	4.0	1,157	0.5	154	1.64 (1.50–1.80)	
<1 year since last use (0.5 years)	1,764	3.6	63	0.3	6	1.42 (1.08–1.86)	
1–4 years since last use (2 years)	5,931	3.0	176	0.4	21	1.23 (1.04–1.46)	
≥5 years since last use (7 years)	3,813	2.4	92	0.3	13	1.07 (0.85–1.34)	
							
Duration of use among current users of HRT (median)							χ^2^_1 _(trend) = 2.1, *p* = 0.2
<1 year (0.5 years)	1,935	3.9	75	0.3	5	1.41 (1.10–1.81)	
1–4 years (3 years)	9,790	4.2	410	0.6	54	1.58 (1.40–1.79)	
5–9 years (6 years)	11,280	3.9	441	0.6	71	1.74 (1.53–1.97)	
≥10 years (11 years)	4,963	3.9	193	0.4	21	1.74 (1.46–2.06)	
							
Duration of use among past users of HRT (median)							χ^2^_1 _(trend) = 8.4, *p* = 0.004
<1 year (0.5 years)	4,014	2.4	95	0.3	13	0.95 (0.77–1.18)	
1–4 years (3 years)	5,152	2.8	143	0.3	17	1.29 (1.07–1.56)	
≥5 years (8 years)	3,061	3.4	104	0.5	14	1.54 (1.23–1.93)	

^a^Numbers do not always add up to total due to missing values. ^b^Stratified by screening centre, age, time since menopause, previous screening, body mass index and previous breast operation. ^c^Reference group. CI, confidence interval; HRT, hormone replacement therapy; RR, relative risk.
